# Influence of thickness and translucency of lithium disilicate ceramic on degree of conversion of resinous materials

**DOI:** 10.4317/jced.56921

**Published:** 2020-08-01

**Authors:** Priscila-Christiane-Suzy Liporoni, Alcira-Cinthia-Rodríguez Ponce, Maiara-Rodrigues de Freitas, Rayssa-Ferreira Zanatta, Maria-Clara-Santos Pereira, Anderson Catelan

**Affiliations:** 1 Assistant Professor, Department of Dentistry, University of Taubaté, Taubaté, SP, Brazil; 2 Master Degree Student, Department of Dentistry, University of Taubaté, Taubaté, SP, Brazil; 3 Undergraduate Student, Department of Dentistry, University of Taubaté, Taubaté, SP, Brazil; 4 Assistant Professor, Graduate Program in Dentistry, Faculty of Health Sciences, University of Western São Paulo, Presidente Prudente, SP, Brazil

## Abstract

**Background:**

In this study was assessed the degree of conversion (DC) of amine-free resin cements light cured through lithium disilicate-reinforced ceramics at different thicknesses and translucency.

**Material and Methods:**

Specimens were divided into 21 groups (n = 5) according to luting agent used: Variolink Esthetic LC (Light shade), RelyX Ultimate (A1 shade), and Filtek Z350 XT Flow (A1 shade); the ceramic translucency: low (LT) and high (HT); and the ceramic thickness: no ceramic (control), 0.5 mm, 1 mm, and 2 mm. A Teflon mold with (5 x 5 x 0.5 mm) was used to standardize the cement and over it the ceramic block from each group was placed. Set was cured using a polywave LED light (1200 mW/cm2 - Bluephase G2) for 40 s. FTIR spectra of uncured and cured materials was obtained and DC calculated from the height of the peaks 1610 and 1640 cm-1. Data were submitted to ANOVA followed by Tukey’s test (α = 0.05).

**Results:**

There was a significant difference for luting agents (*p*< 0.0001) and translucency (*p* = 0.025), but not for thickness (*p* = 0.73). Dual amine-free RelyX Ultimate showed the lowest DC values and higher translucency promoted higher DC.

**Conclusions:**

Dual amine-free cement showed the lowest monomer conversion and higher translucency ceramics promoted a higher DC.

** Key words:**Ceramic, degree of conversion, resin cement, thickness, translucency.

## Introduction

Metal-free ceramic restorations such as veneers, inlays, onlays, and crowns have been routinely used in clinical practice (1,2), mainly due to its good optical, physical, chemical, and biological properties (3,4). However, success of these restorations is highly dependent of an effective adhesion between ceramic, resin cement, and dental tissues (5-8).

Resin cement is usually the choice for bonding metal-free indirect esthetic restorations (3,4,8,9) and its polymerization modes can be divided in chemical, physical (light curing), and dual, which combines the desirable properties from physical and chemical activation (4,6,10,11). Dual system has been developed to compensate the polymerization deficiencies in attenuated light or its absence, enabling its indication in different clinical situations, resulting in superior properties compared to chemically activated cements (6,12).

Polymerization reaction of dual cements occurs physically by reaction between tertiary amines and monomers, and chemically by mixing a catalyst paste (benzoyl peroxide) with a base paste (tertiary amine) (4,12). As tertiary amines are not all consumed during the polymerization reaction in chemical and dual systems, there is a tendency to stain or darken over time due to its oxidation, which can lead to esthetic impairment, especially in case of veneers (2,4,12).

In order to minimize this problem, amine free resin cements have been recently introduced in market to promote the esthetic longevity of indirect restorations (13,14). Flowable composite can also be used (8), but these systems have physical activation only, and it is estimated that the light irradiance emitted by light curing unit that reaches the cement is reduced when it passes through the ceramic, reducing the degree of conversion (DC) and consequently the mechanical properties of material, which may affect the durability of indirect restorations (6,11,13,15,16). This event is dependent on light intensity, curing unit, ceramic thickness, opacity, among others (5,9,13,15,17).

The aim in this study was to evaluate the DC of light-cured tertiary amine-free luting agents through lithium disilicate-reinforced ceramics with different thickness and translucency. The null hypothesis tested was that there would no change on DC of polymerized resin-based material through different ceramic thickness and translucency.

## Material and Methods

-Obtaining specimens

Resinous specimens were made using a square Teflon mold with 5 mm side and 0.5 mm thickness. Luting agents tested were: a light cure resin cement, VE - Variolink Esthetic LC (shade Light+, Ivoclar Vivadent, Schaan, Liechtenstein); a dual resin cement, RXU - RelyX Ultimate (shade A1, 3M ESPE, St Paul, MN, USA); and a light cure flowable composite, FZ350 - Filtek Z350 XT Flow (shade A1, 3M ESPE).

Teflon mold was filled with the resinous material, followed by the placement of a Mylar strip over it and removal of excess. Then, a low (LT) or high (HT) translucency lithium disilicate-reinforced ceramic (IPS e.max CAD) was positioned over the cementing agent, and the set was cured with a polywave LED unit (Bluephase G2 - Ivoclar Vivadent) for 40 s at irradiance of 1200 mW/cm2. Three different ceramic thicknesses were tested: 0.5 mm, 1.0 mm or 2.0 mm. The control groups for each cement agent were made from the direct light curing, without the interposition of ceramic.

Thus, 21 groups (n = 5) were formed. Specimens were stored dried in aluminum foil covered flasks at 37ºC for 24 h, for further DC analysis.

-Degree of conversion (DC) calculation

The DC was obtained by Fourier Transformed Infrared Spectroscopy (FTIR - Spectrum 100 FTIR/ATR; Perkin Elmer, Waltham, MA, USA). Unpolymerized material spectrum was obtained in the region between 650 to 4000 cm-1 with 16 scans and 4 cm-1 resolution. Following, the polymerized material spectrum was obtained with the same parameters described. Baseline correction was performed in region between 1590 to 1660 cm-1, for observations at 1610 and 1640 cm-1, indicating, respectively, the aromatic bisphenol and aliphatic vinyl bonds of methacrylate functional group. DC was calculated from following equation: DC = 100 x [1- (cured (1640cm-1/1610 cm-1)/Uncured (1640cm-1/1610 cm-1))].

-Statistical analysis

Data were checked for normality and homogeneity, and then analyzed by three-way ANOVA followed by Tukey’s test at significance level of 5%. To compare the control group with the experimental groups within each luting agent, Dunnett’s statistical test (α = 0.05) was used.

## Results

Three-way ANOVA test showed a significant difference only for luting agents (*p* < 0.0001) and translucency (*p* = 0.0252). For the thickness factor (*p* = 0.267), and for interaction between factors was no significant (*p* = 0.730). Regarding the materials, RXU resin cement showed the lowest DC compared to FZ350 and VE, which showed no significant difference between them. Higher translucency promoted a higher DC compared to lower translucency (Table 1).

**Table 1:**
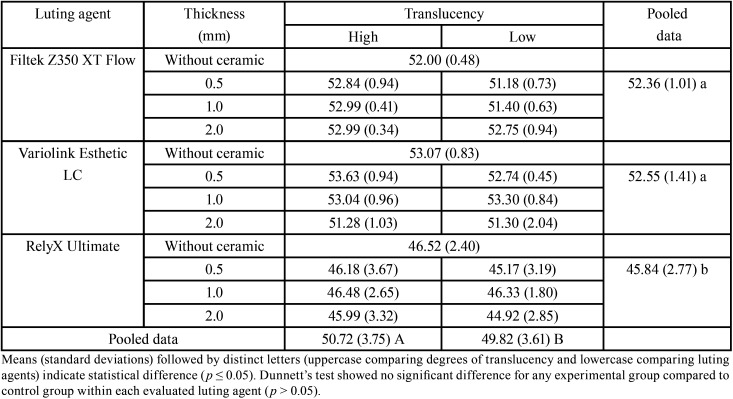
Degree of conversion (%) values according to the luting agent, thickness, and translucency of ceramic.

## Discussion

For success and longevity of ceramic restorations a correct adhesive cementation protocol is essential. Therefore, it is necessary to obtain adequate physicochemical properties of resin cement bonding agents, and since ceramics with different degrees of translucency and thickness are used in clinical practice, there is differences at irradiance that reaches the resin cement or flowable composite (18).

Recently, it has been reported a yellowish or unpleasant shade alteration in indirect veneers and its one of the major esthetic problems reported in case of long-term ceramic veneers (2,4,12). This is mainly attributed to the oxidation of tertiary amine presented in resin cements composition, and therefore flowable composites have been used instead of resin cements containing tertiary amine, to overcome this problem.

During cementation of indirect restoration, the irradiance of light curing unit arriving at the luting agent is impaired as the ceramic part is more opaque and thicker, which may reduce the physicochemical properties of these materials (9,12,19). However, in this study there was no difference on DC for any of the luting agents evaluated when light cured under the different thickness of high and low translucent ceramic blocks. All experimental groups presented similar DC values to their control group, in which the cementing agent was cured without the interposition of ceramic block.

For the highest ceramic thickness tested, 2 mm, the DC was not reduced. This is probably due to high irradiance of curing device combined with the thin thickness of resin cementing agent, which provided the adequate values obtained regarding the DC. It is also important to highlight the use of a compatible spectrum curing unit with the absorption wavelength of alternative photoinitiator to camphorquinone present in luting agents used, making possible an adequate polymerization of material.

Regarding the translucency, it has been reported that the higher the opacity, as well as the higher saturation, the lowest transmission of light source to cementing agent, which results in lower physicochemical properties (3,13-15). As observed in this research, the opaquer lithium disilicate ceramics showed lower DC during cementation compared to more translucent ceramic. Lower translucency promotes light dissipation of light curing unit and lower light intensity reaches to cementing agent (13,15). Thus, in the case of darker teeth rehabilitation, where the use of higher opacity ceramics is required, it has been recommended to increase the light curing time (13).

In comparison between cementing agents, RXU resin cement showed a lower DC compared to other resinous materials, corroborating the findings of a previous study (20). It also evaluated the crosslink density and Knoop hardness, which showed higher values than other resinous materials, suggesting a higher polymerization rate for RXU cement (20), since larger amounts of free radicals promote more growth centers of polymeric network (21,22). On the other hand, the faster formation of the crosslinked polymeric network ends up limiting the free radical mobility (23), which causes the formation of microgels (unreacted monomers and oligomers) between high density crosslinked zones, resulting in a heterogeneous polymer (21) and may have contributed to lower monomer conversion.

Light curing unit irradiance, exposure time, light transmission by ceramics, and the light absorption length of photoinitiator of resin cement are factors that significantly affect the conversion of monomers to polymers. Since adequate polymerization is fundamental to obtain physical, chemical, and biological properties, exclusively light curing resin cement without the amine presence in its composition, due to higher color stability, it seems to be a viable alternative for cementing higher translucent glass ceramics up to 2-mm thick, provided that curing is performed using high irradiance.

## Conclusions

Regarding the luting agent, dual amine-free resin cement showed lower conversion of monomers to polymers. In relation to translucency, the ceramic with higher translucency promoted the highest degree of conversion in all cementing agents. Finally, in relation to ceramic thickness interposed during light curing, it did not influence the degree of conversion of resinous materials when cured using an appropriate irradiance and wavelength.
